# Development of key performance indicators for renal replacement therapy in adult intensive care to guide safe and cost-effective therapy

**DOI:** 10.1186/cc13586

**Published:** 2014-03-17

**Authors:** A Fischer, S Finney

**Affiliations:** 1 Royal Brompton Hospital, London, UK

## Introduction

Renal replacement therapy (RRT) is common. We undertook to develop and report some key performance indicators (KPIs) to monitor our provision of this costly therapy. We utilised data already collected in an electronic clinical information system that records the care received by our patients. We reduced our prescribed RRT dose to 25 ml/kg/hour in December 2011 following an appraisal of the literature [1]. We assessed whether the KPIs informed us if our practice changed and such changes were sustained.

## Methods

We calculate the hourly effluent rate corrected for a patient's predicted body weight, and the lifespan of haemofilters. This takes less than 30 minutes each month. Statistical process control charts (SPCs) are used to assimilate the indicators over time.

## Results

**Figure 1:**
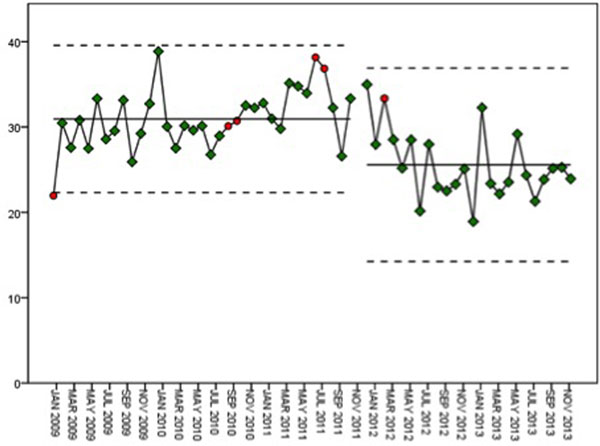
**Delivered dose of RRT each month**.

A total of 736 patients received RRT during the study. Prior to the dose change, the mean set and delivered doses were 39 and 31 ml/ kg/hour respectively. Thereafter the mean set and delivered doses were 33 and 26 ml/kg/hour respectively. Whilst higher than our guideline dose, they are significantly less than the doses prior to the change in practice (both *P *< 0.001). The SPC indicates that the change in practice has been sustained. See Figure 1 and Table 1.

**Table 1:** KPI for renal replacement therapy 2013

	Jan 2013	Feb 2013	Mar 2013	Apr 2013	May 2013	Jun 2013	Jul 2013	Aug 2013	Sep 2013	Oct 2013	Nov 2013	Dec 2013
AICU-RBH no. of patients on CVVHD	**8**	**8**	**17**	**15**	**6**	**7**	**13**	**12**	**9**	**15**	**21**	
AICU-RBH no. of patient-days on CWHD	61	73	98	86	65	85	44	77	112	119	137	
AICU-RBH filter started	32	30	52	47	34	50	26	60	71	62	74	
AICU-RBH hours of CWHD	1,154	1,463	1,765	1,532	1,317	1,565	693	1,422	2,215	2,157	2,393	
AICU-RBH estimated no bags	544	602	763	689	636	695	294	629	995	952	1,054	
AICU-RBH fluid cost (£)	4,994	5,526	7,004	6,325	5,838	6,380	2,699	5,774	9,134	8,739	9,676	
AICU-RBH ml/kg/hour actual	39.90	28.17	29.10	30.53	33.93	31.52	31.25	29.90	30.40	32.55	32.36	
AICU-RBH ml/kg/hour 24 hours	32.46	23.44	22.23	23.60	29.32	24.43	21.34	23.93	25.25	25.38	24.01	
AICU-RBH percent on CWHD F	0.79	0.84	0.75	0.67	0.84	0.77	0.66	0.77	0.82	0.76	0.73	
AICU-RBH average filter life (hours)	36.06	48.77	34.02	32.60	38.74	31.30	26.65	23.70	31.20	34.79	32.34	
AICU-RBH filter cost (£)	2,477	2,322	4,025	3,638	2,632	3,870	2,012	4,644	5,495	4,799	5,728	
AICU-RBH effluent bags cost (£)	680	753	954	861	795	869	368	786	1,244	1,1 90	1,318	
AICU-RBH total consumable (£)	3,157	3,075	4,979	4,499	3,427	4,739	2,380	5,430	6,739	5,989	7,045	
AICU-RBH total cost CWHD (£)	**8,151**	**8,601**	**11,983**	**10,824**	**9,265**	**11,119**	**5,079**	**11,204**	**15,873**	**14,728**	**16,721**	
AICU-RBH average cost per patient (£)	1,019	1,075	705	722	1,544	1,588	391	934	1,764	982	796	
AICU-RBH average cost per patient day (£)	134	118	122	126	143	131	115	146	142	124	122	

## Conclusion

The KPIs could be produced quickly and allowed monitoring of the reduction in RRT dosing, assuring us that it is in excess of 20 ml/ kg/hour. The KPIs did not require additional data collection processes. We are developing similar indicators for other organ systems, therapies and processes.
